# Low carbohydrate diet-based intervention for obstructive sleep apnea and primary hypothyroidism in an obese Japanese man

**DOI:** 10.1186/s12930-016-0029-8

**Published:** 2016-08-05

**Authors:** Yoshio Tokuchi, Yayoi Nakamura, Yusuke Munekata, Fumio Tokuchi

**Affiliations:** 1 Tokuchi Naika Clinic, 3-6, Iwamizawa, Hokkaido 068-0023 Japan; 2 Hokkaido Air Water Inc., Kikusui 5-2-3-12, Shiroishi-ku, Sapporo, Hokkaido 003-0805 Japan

**Keywords:** Obstructive sleep apnea, Continuous positive airway pressure, Low-carbohydrate diet, Obesity, Hypothyroidism, Levothyroxine replacement

## Abstract

**Background:**

Obesity is a major risk factor for obstructive sleep apnea (OSA), and weight loss is necessary in the overall management of obese patients with OSA. However, primary care physicians can provide only limited weight loss with lifestyle interventions, usually reducing a patient’s body weight by only 2.5 kg or less after 6–18 months.

**Case presentation:**

A 45-year-old Japanese man was referred to our clinic owing to obesity, daytime sleepiness, and snoring during sleep. His weight was 130.7 kg and his body mass index (BMI) was 41.0 kg/m^2^. He underwent polysomnography, which revealed OSA with an apnea–hypopnea index of 71.2 events/h (normal, <5 events/h). His laboratory results were as follows: thyroid stimulating hormone, >500 μIU/mL; free triiodothyronine, 1.4 pg/mL; free thyroxine, <0.15 ng/dL; thyroid peroxidase antibody, 10 IU/mL; thyroglobulin antibody, >4000 IU/mL; total cholesterol (TC), 335 mg/dL; high-density lipoprotein cholesterol, 45 mg/dL; triglycerides (TGs), 211 mg/dL; low-density lipoprotein cholesterol, 248 mg/dL; fasting blood sugar, 86 mg/dL; and glycated hemoglobin (HbA1c), 6.1 %. These results showed that he also had primary hypothyroidism (Hashimoto’s disease). Continuous positive airway pressure (CPAP), levothyroxine replacement, and a low-carbohydrate diet (LCD) were initiated. CPAP use and a euthyroid condition induced by 175 μg/day levothyroxine allowed the patient to proactively reduce his body weight. After 18 months, the patient achieved a weight reduction of 32.4 kg (25 % of his initial weight) and a BMI reduction of 10.2 kg/m^2^, as well as improved laboratory results, including an HbA1c level of 5.3 %, TC level of 194 mg/dL, and TG level of 89 mg/dL.

**Conclusion:**

An LCD may be an effective intervention for weight loss in obese Japanese patients with OSA. Further studies are needed to investigate the weight loss effect of an LCD compared with a conventional calorie-restricted diet. Hopefully, this case report will help to improve the management of obese Asian patients with OSA who typically consume a higher amount of carbohydrates.

## Background

Obesity is a major risk factor for obstructive sleep apnea (OSA) [1], and weight loss is necessary in the overall management of obese patients with OSA. However, primary care physicians can provide only limited weight loss by medical therapy alone. Previous trials, in which primary care physicians offered lifestyle counseling, achieved a weight loss of only 2.5 kg or less after 6–18 months [2, 3]. In addition, intensive medical therapy or enhanced weight loss counseling for patients with obesity has been shown to achieve a mean weight loss of 4.3–5.1 kg after 2–3 years [1, 2, 4]. In contrast, continuous positive airway pressure (CPAP)—the standard treatment for OSA—may actually increase the body weight of patients [5]. On the other hand, hypothyroidism can be associated with OSA [6], and some symptoms, such as daytime sleepiness, fatigue, and lethargy, overlap for both diseases.

Here, we describe an obese Japanese man with OSA and primary hypothyroidism who was treated with CPAP, levothyroxine replacement, and a low-carbohydrate diet (LCD) and achieved a weight reduction of 32.4 kg (25 % of his initial weight) and a body mass index (BMI) reduction of 10.2 kg/m^2^ after 18 months.

## Case presentation

In January 2014, a 45-year-old Japanese man was referred to our clinic owing to obesity, daytime sleepiness, and snoring during sleep. His Epworth Sleepiness Scale score was 10 points. He underwent overnight diagnostic polysomnography in February 2014. Upon admission, his height was 178.6 cm, weight was 130.7 kg, abdominal circumference was 122 cm, and BMI was 41.0 kg/m^2^. Physical examination showed that the thyroid gland was not palpable. His serum levels of creatine kinase and creatinine were elevated at 1581 U/L and 1.46 mg/dL, respectively, while his other laboratory results were as follows: aspartate aminotransferase, 57 U/L; alanine aminotransferase, 49 U/L; lactate dehydrogenase, 353 U/L; total cholesterol (TC), 335 mg/dL; high-density lipoprotein cholesterol, 45 mg/dL; triglycerides (TGs), 211 mg/dL; low-density lipoprotein cholesterol, 248 mg/dL; fasting blood sugar, 86 mg/dL; glycated hemoglobin (HbA1c), 6.1 %; thyroid-stimulating hormone (TSH), >500 μIU/mL; free triiodothyronine, 1.4 pg/mL; free thyroxine, <0.15 ng/dL; thyroid peroxidase antibody, 10 IU/mL; and thyroglobulin antibody, >4000 IU/mL (Table 1). Blood samples were evaluated using routine methods at Sapporo Clinical Laboratory Inc. (Sapporo, Hokkaido, Japan). Based on these results, indicating hypothyroidism and thyroglobulin antibody positivity, the patient was diagnosed with severe primary hypothyroidism (Hashimoto’s thyroiditis). His dyslipidemia and impaired glucose tolerance were considered as secondary changes due to primary hypothyroidism and obesity. Because polysomnography showed an apnea–hypopnea index (AHI) of 71.2 events/h (normal, <5 events/h) and a minimal SpO_2_ of 60 %, he also was diagnosed with severe OSA associated with autoimmune hypothyroidism. Therefore, in the beginning of March 2014, nasal CPAP (ICON+Auto, auto-adjusting CPAP; Fisher & Paykel Healthcare Limited, Auckland, New Zealand; minimal pressure, 4.0 cmH_2_O; maximal pressure, 18.0 cmH_2_O) and low-dose levothyroxine (12.5 μg/day) were initiated. Upon initiation of the CPAP therapy, the patient was informed that he would have to continue with the CPAP therapy unless his body weight decreased sufficiently.

**Table 1:** Laboratory results of blood samples on admission

CBC	
WBCs	5500 × 10^3^/μL
RBCs	452 × 10^4^/μL
Hgb	14.1 g/dL
PLTs	25.6 × 10^4^/μL
Glucose	
FBS	86 mg/dL
HbA1c	*6.1* %
Lipids	
TC	*335* mg/dL
HDL-C	45 mg/dL
TGs	*211* mg/dL
LDL-C	*248* mg/dL
Thyroid	
TSH	*>500* μIU/mL
fT4	*<0.15* ng/dL
fT3	*1.4* pg/mL
Tg antibody	*>4000* IU/mL
TPO antibody	10 IU/mL
Biochemistry	
TP	6.9 g/dL
TB	0.8 mg/dL
AST	*57* U/L
ALT	*49* U/L
LDH	*353* U/L
ALP	209 U/L
γGTP	75 U/L
CK	*1581* U/L
Cr	*1.46* mg/dL
UA	6.9 mg/dL
BUN	11.4 mg/dL
Na	139 mEq/L
K	4.1 mEq/L
Cl	98 mEq/L
CRP	0.17 mg/dL

Abnormal findings are italicized

*ALP* alkaline phosphatase, *ALT* alanine aminotransferase, *AST* aspartate aminotransferase, *BUN* blood urea nitrogen, *CBC* complete blood count, *CK* creatine kinase, *Cl* chloride; *Cr* creatinine, *CRP* C-reactive protein, *FBS* fasting blood sugar, *fT3* free triiodothyronine, *fT4* free thyroxine, *γGTP* gamma-glutamyl transferase, *HbA1c* glycated hemoglobin, *HDL*-*C* high-density lipoprotein cholesterol, *Hgb* hemoglobin, *K* potassium, *LDH* lactate dehydrogenase, *LDL*-*C* low-density lipoprotein cholesterol, *Na* sodium, PLTs platelets, *RBCs* red blood cells, *TB* total bilirubin, *TC* total cholesterol, *Tg* thyroglobulin, *TGs* triglycerides, *TP* total protein, *TPO* thyroperoxidase, *TSH* thyroid-stimulating hormone, *UA* uric acid, *WBCs* white blood cells

In April 2014, the patient’s body weight had decreased to 125.2 kg, and post-CPAP polysomnography indicated improved OSA (AHI, 2.8 events/h; minimal SpO_2_, 88 %). A monthly follow-up visit was scheduled to check his body weight and adherence to CPAP. The memory stick of the CPAP machine showed that he used CPAP for an average of 6 h per night, 6 days a week, and that he had an AHI of approximately 2.0 events/h. In the course of gradually increasing the dose of levothyroxine, the patient reportedly felt better and had recovered from his fatigue and lethargy.

To address his weight loss as a priority, an LCD was initiated in July 2014 [7]. Yamada et al. [8] reported the benefits of a non-calorie-restricted LCD with the amount of carbohydrates limited to 70–130 g/day for Japanese patients with type 2 diabetes. The patient consumed 50 g of white rice per meal in addition to vegetables, fish, eggs, and meat in the morning and at noon; however, he did not eat rice, noodles, or other carbohydrates at night. In February 2015, the dose of 175 μg/day levothyroxine appeared to maintain a euthyroid state (TSH, 0.75 μIU/mL), and his other laboratory results were within normal limits, including an HbA1c level of 5.3 %, TC level of 194 mg/dL, and TG level of 89 mg/dL.

**Fig. 1:**
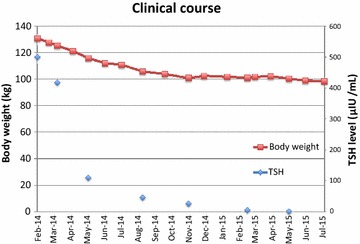
Changes in the patient’s body weight and thyroid-stimulating hormone (TSH) level over time

Finally, in July 2015, the patient’s body weight reached 98.3 kg, a reduction of 32.4 kg (25 % of his initial body weight), while his BMI decreased from 41.0 to 30.8 kg/m^2^. Figure 1 shows changes in his body weight and TSH level over time. The patient felt motivated by the prospect of discontinuing the CPAP therapy, and was encouraged by the consistent weight loss at the monthly clinical visits. He also wanted to continue with the LCD because he did not find it difficult.

## Discussion

The course of this patient provided two important clinical suggestions. First, this obese Japanese man with OSA achieved a weight loss of 32.4 kg (25 % of his initial body weight) by medical therapy alone. Second, an LCD may be an effective intervention for obese Japanese patients with OSA.

There are three potential mechanisms for this patient’s successful weight loss. First, CPAP was able to control his sleepiness effectively. Second, his primary hypothyroidism was corrected to a euthyroid state with 175 μg/day levothyroxine. Third, an LCD was initiated as dietary therapy. Therefore, both OSA and primary hypothyroidism were treated simultaneously in this patient. He recovered from his fatigue and lethargy, thus enabling him to reduce his body weight more proactively. In developed Western countries, bariatric surgery can be performed for weight loss in patients with obesity [1, 4]. Such surgeries have been shown to achieve a weight loss of 24.5–27.8 kg (20.6–24.5 % of initial body weight) after 2–3 years [1, 4]. Therefore, the weight loss of this patient after 2 years was similar to that observed in those who have undergone bariatric surgery.

An LCD is an effective intervention for weight loss in patients with obesity [7]. Japanese individuals typically consume a higher amount of carbohydrates compared with Western populations, obtaining approximately 60 % of their total energy intake from carbohydrates [9]. Therefore, an LCD may have a superior weight loss effect in obese Japanese patients. As far as we know, this is the first case in which an LCD was initiated in an obese Japanese patient with OSA. An LCD was introduced in this case in July 2014. His weight loss from June to July was 1.4 kg, whereas, from July to August, his weight loss was 5.1 kg. Thus, the LCD seemed to accelerate his weight loss. His glucose and lipid metabolism also improved without diabetes or hyperlipidemia medication. In a previous large long-term observational study in Japan, a usual LCD was found to be significantly inversely associated with cardiovascular and total mortality [9]. Therefore, an LCD could allow Japanese primary care physicians to achieve clinically meaningful weight loss in their obese patients, which they might not be able to accomplish otherwise. However, because CPAP may increase the body weight of obese patients with OSA [5, 10], additional therapy for weight loss is required. Furthermore, weight loss after levothyroxine replacement for hypothyroidism does not always occur [11, 12]. In a previous study, levothyroxine achieved a weight loss of only 4.3 kg (from an average of 83.7–79.4 kg) after 1 year [13]. Moreover, such weight loss occurred due to decrease in lean body mass, whereas fat mass did not decline [13]. This suggests that the weight loss after levothyroxine replacement was due to loss of excess body water associated with untreated myxedema [13]. Thus, levothyroxine replacement alone may not achieve an adequate weight loss in patients with hypothyroidism.

## Conclusion

We described an obese Japanese man with OSA and primary hypothyroidism who achieved a weight loss of 32.4 kg (25 % of his initial body weight) by medical therapy alone. We further demonstrated that an LCD may be an effective intervention for weight loss in obese Japanese patients with OSA. Further studies are needed to investigate the weight loss effect of an LCD compared with a conventional calorie-restricted diet. Hopefully, this case report will help to improve the management of obese Asian patients with OSA who typically consume a higher amount of carbohydrates.

## Abbreviations

AHI: apnea–hypopnea index
BMI: body mass index
CPAP: continuous positive airway pressure
HbA1c: glycated hemoglobin
LCD: low-carbohydrate diet
OSA: obstructive sleep apnea
TC: total cholesterol
TGs: triglycerides
